# Shared biomarkers and immune cell infiltration signatures in ulcerative colitis and nonalcoholic steatohepatitis

**DOI:** 10.1038/s41598-023-44853-6

**Published:** 2023-10-28

**Authors:** Wenxin Wang, Xin Gao, Ning Kang, Chen Wang, Chenyang Li, Huan Yu, Xiaolan Zhang

**Affiliations:** Department of Gastroenterology and Hepatology, Hebei Key Laboratory of Gastroenterology, Hebei Institute of Gastroenterology, Hebei Clinical Research Center for Digestive Diseases, The Second Hospital of Hebei Medical University, Shijiazhuang, 050000 Hebei China

**Keywords:** Biomarkers, Gastroenterology

## Abstract

The coexistence of ulcerative colitis (UC) and nonalcoholic steatohepatitis (NASH) involves a intricate interplay, though the precise pathophysiological mechanisms remain elusive. To shed light on this, our study endeavors to unravel the shared gene signatures and molecular mechanisms by employing quantitative bioinformatics analysis on a publicly available RNA-sequencing database. Gene expression profiles of UC (GSE87466) and NASH (GSE89632) were retrieved from the Gene Expression Omnibus (GEO) database. Differentially expressed genes (DEGs) were analyzed using R software. After identifying common DEGs, functional enrichment analysis, protein–protein interaction (PPI) network analysis and module construction were performed to obtain candidate hub genes. GSE47908 for UC and GSE159676 for NASH were selected to validate the obtained candidate genes. A total of 119 common DEGs were found in NASH and UC patients. Functional and pathway analyses emphasized that viral infection, inflammation and immune response were enriched in these two diseases. After module construction and validation, CD2, CD8A, GNLY, IFI44, NKG7 and OAS2 were identified as hub genes. 6 hub genes and their combined prediction scores were found with an impressive accuracy and sensitivity. Functional estimation, gene set enrichment analysis and immune infiltration signature identification showed notable associations of the six hub genes with T cells, natural killer cells and type I interferon levels. In addition, we constructed UC combined with NASH mice model successfully with significantly higher expression of hub genes in both liver and colonic tissues than those in control group. Our study elucidates 6 hub genes of UC and NASH, which may participate in immune, inflammatory and antiviral effects. These findings provide some potential biochemical markers for further exploration of UC coexistence with NASH.

## Introduction

Ulcerative colitis (UC) is one of the predominant forms of inflammatory bowel disease (IBD), which is characterized by relapsing and remitting inflammation of the intestine, starting in the rectum and extending to proximal segments of the colon^1^. The etiology of UC remains unclear, but the incidence of UC is increasing yearly worldwide. Approximately 20% of patients may have acute exacerbation of the disease during treatment, accompanied by a variety of extraintestinal manifestations, which has a negative impact on the quality of life, social function and mental health of patients.

Nonalcoholic fatty liver disease (NAFLD) accounts for approximately 40% of liver disorders. NAFLD is a complex chronic liver disease closely related to overweight, obesity, insulin resistance and metabolic dysfunction. It is characterized by steatosis of hepatocytes, and the disease affects approximately 25% of the world's population^2,3^. Nonalcoholic steatohepatitis (NASH), as a progressive form of NAFLD, is characterized by histological features of at least 5% steatosis, the presence of hepatocyte ballooning degeneration and lobular inflammation with or without fibrosis and has the possibility of developing into cirrhosis and liver cancer. However, due to the complexity of NASH, the pathogenesis is still not completely clear, and there is no effective treatment widely used in clinical practice.

In the investigation of IBD, hepatobiliary manifestations and steatohepatitis are some of the most common extraintestinal manifestations. UC patients are at increased risk of developing NAFLD^4,5^. In addition, the presence of NAFLD can further complicate the clinical management of IBD, posing additional challenges. It is worth noting that the mortality rate among hospitalized IBD patients with liver disease is twice as high compared to those with IBD alone. Nevertheless, the mechanisms that underlie this phenomenon necessitate further investigation.

Some interesting concepts are being proposed while studying the complex connection between NASH and UC. "Gut-liver axis" reflects gut microbiota dysbiosis, mucosal barrier disruption or intestinal permeability increase the development and disease progression of chronic liver disease through the immune-mediated inflammatory response, and chronic liver disease itself aggravates intestinal disorders^6^^.^ Our previous research found that experimental models of colitis activate hepatic stellate cells and TLR4 signaling through the gut-liver axis to aggravate liver fibrosis, verifying the important relationship between the gut and liver diseases^7^. These concepts partially explain the susceptibility of UC to merge with NASH. However, at the molecular level, our understanding of the fundamental signals when both diseases coexist is still limited. This lack of knowledge may be a crucial factor influencing future clinical diagnosis and treatment.

In our present study, we explored the common hub genes of UC and NASH and estimated pathways in which they participate through bioinformatics analysis of data in a public database. In addition, the expression of these hub genes was validated by UC combined with NASH mice model. These findings may help to identify underlying common pathophysiological mechanisms and potential biomarkers of UC coexistence with NASH.

## Materials and methods

### Dataset collection and DEG identification

We used ulcerative colitis and nonalcoholic steatohepatitis as keywords to retrieve gene expression profiles and corresponding clinical data from the Gene Expression Omnibus (GEO) database (http://www.ncbi.nlm.nih.gov/geo). The arrays containing at least 15 samples in each group were selected, and two datasets were finally included in this study, GSE87466 and GSE89632, which were selected for RNA sequencing analysis. The GSE87466 dataset contains RNA sequencing results of intestinal mucosal tissues from 87 patients with UC and 21 normal controls. Liver biopsy sequencing results were obtained from 19 NASH patients and 24 healthy control patients in the GSE89632 dataset.

Then, the limma package (Version 3.42.2) in R (Version 4.0.3) software was used to normalize the data and perform differential analysis, and the fold changes (FCs) were calculated for individual gene expression. The differentially expressed genes (DEGs) were defined as those with |log_2_FC|> 0.585 and an adjusted *P* value < 0.05. The common DEGs between UC and NASH were obtained by the ggplot2 package (Version 3.3.3) and ComplexHeatmap package (Version 2.2.0) of R software for visualization.

### Functional enrichment analysis

To further reveal the common trends of DEGs, we used the clusterProfiler package of R software to perform enrichment analysis^8^. The enrichment analysis was performed using the Gene Ontology (GO) and Kyoto Encyclopedia of Genes and Genomes (KEGG) databases. The GO database can be used to obtain the correlation of target genes, and functions were further divided into biological process (BP), cellular component (CC) and molecular function (MF) terms. The KEGG database was mainly used for bioinformatics analysis at the pathway level^9^.

### Protein–protein interaction (PPI) network analysis and hub gene identification

Based on the obtained common DEGs, the Search Tool for the Retrieval of Interacting Genes Database (STRING) was used to construct the PPI network to determine how proteins encoded by these DEGs interact with each other (https://cn.string-db.org/^10^. The interaction score was set to high confidence (confidence > 0.7). The PPI network was visualized by Cytoscape (Verson 3.9.1), and molecular complex detection (MCODE), a plugin of Cytoscape, was used to identify the core functional genes from the network. The options of MCODE were set as follows: Degree Cutoff in Network Scoring was set as 2, Node Score Cutoff in Cluster Finding was set as 0.2, K-Core was set as 2 and Max. Depth was set as 100. Meanwhile, the cytoHubba plugin was used to measure the core genes in different topological analysis methods to explore the important genes in the PPI network, of which the maximal clique centrality (MCC), maximum neighborhood component (MNC), density of maximum neighborhood component (DMNC), edge percolated component (EPC) and Degree are the five most commonly used topology analysis methods. The overlap of genes in the cluster with a score > 3 measured by MCODE and the top 20 DEGs measured by cytoHubba were determined as hub genes.

### Validation and diagnostic features of hub gene expression

GSE47908 and GSE159676 were selected to validate the obtained hub genes. GSE47908 contained colon biopsies from 45 UC patients and 15 controls, while GSE47908 contained liver tissue samples from seven NASH patients and five healthy controls. The Mann‒Whitney U test was used to verify the hub genes in the two datasets. Subsequently, logistic regression analyses were applied to estimate the joint predictive score, and a receiver operating characteristic (ROC) curve was used to assess the accuracy of the diagnostic features of the hub genes and joint predictive score.

### Functional estimation of hub genes and integrative analysis of immune infiltration

Functional analysis of hub genes and their interacting genes was performed through GeneMANIA (http://genemania.org/), which can predict correlations, including protein and genetic interactions, pathways, coexpression, colocalization and protein domain similarity. Then, the immune infiltration associated with UC and NASH was analyzed using a single sample gene set enrichment analysis (ssGSEA) through ImmuCellAI (http://bioinfo.life.hust.edu.cn/ImmuCellAI/#!/), a tool capable of estimating the levels of 24 immune cells from gene expression datasets. Then, correlations between hub genes and immune cells were examined using Spearman correlation analysis to reveal the relationship between the obtained hub genes and immune function, and the results were visualized by ggplot2 package and ComplexHeatmap package of R software.

### GSEA analysis of hub genes

Gene set enrichment analysis (GSEA) was performed using GSEA V4.3.2 software. The patients were divided into those with high-expression and low-expression of hub genes, and GSEA was performed to identify differentially regulated pathways and signaling pathways in UC and NASH.

### Animals and treatments

Male C57BL/6 mice (weight 20–24 g, 8 weeks old) were purchased from Charles River Company Limited (Beijing, China) and maintained in the Animal Experiment Center of the Second Hospital of Hebei Medical University (Shijiazhuang, Hebei, China). All mice were maintained at a temperature of 23 ± 2 °C, 70–75% humidity, 12 h light/alternating conditions, and had freedom of access to food and water. All animal experimental protocols followed the Institutional Animal Care guidelines and were approved by the Laboratory Animal Ethics Committee of the Second Hospital of Hebei Medical University. The study was reported in accordance with the ARRIVE essential ten guidelines.

After one week of adaptation feeding, the mice were randomly divided into two groups. Group one was subjected the methionine choline deficiency diet (MCD) (MolDiets, China, 16 kcal% protein, 63 kcal% carbohydrate, 21 kcal% fat) and concurrently received dextran sulfate sodium (DSS) in drinking water for 5 days followed by distilled water for 2 days in a cyclic manner, totaling 4 weeks, to establish mice model of NASH combined with colitis (DSS + MCD group). Group 2 served as the control group and was provided with the methionine and choline sufficient diet (MCS) and distilled water (Control group). The animal model construction period lasted for 4 weeks.

### Histopathological staining

After the termination of modeling, liver and colon tissue from each group were rapidly separated from mice. The fresh liver tissues were frozen with OCT compound at −18 ℃ (Sorabio, Beijing, China), cut into 5 μm sections, stained with oil red O and re-stained with hematoxylin. Meanwhile, liver and colon tissues were fixed with 4% paraformaldehyde, embedded in paraffin, cut into 4 μm sections and stained with hematoxylin and eosin (H&E).

### Real-time quantitative polymerase chain reaction (RT-PCR) analysis

Total RNA was obtained from liver or colon tissue using the RNA Easy Fast Tissue/Cell Kit (TIANGEN Biotech, Beijing, China) according to the instructions. cDNA was synthesized from total RNA using the FastKing RT Kit (TIANGEN Biotech, Beijing, China). RT-PCR was performed with SuperReal PreMix Plus (SYBR Green) (TIANGEN Biotech, Beijing, China) by ABI StepOnePlus™ RT-PCR detection system (ABI, Connecticut, USA). GAPDH was used as the endogenous control, and the 2^−△△Ct^ method was used to calculate. The primer sequences are shown in Table1.

**Table 1:** Primer sequences for RT-PCR.

Name	Primer sequence (5’ → 3’)	
CD2	F	TTCCTGGGTAGCTTCTTTCTGC
	R	TTGGGGATGTTCAGGGTGATG
CD8A	F	AAGAAAATGGACGCCGAACTT
	R	AAGCCATATAGACAACGAAGGTG
NKG7	F	TCAAGTCCAGACATTCTTCTCCT
	R	CACAAGGTTTCATACTCAGCCC
OAS2	F	CGGGAAACAGCCCTAAGAGG
	R	AGCGTAGAGGATTGAAGACTGG
IFI44	F	GACAAGAGGCATTGCTGTGTT
	R	CGTGTTTGCTGAACCAGGTCT
GAPDH	F	AGGTCGGTGTGAACGGATTTG
	R	TGTAGACCATGTAGTTGAGGTCA

### Statistical analysis

The statistical analyses were performed using R software (Version 4.0.3), GraphPad Prism (Version 9.0.1) and SPSS (Version 26.0.0.0), and *P* < 0.05 was considered statistically significant.

## Results

### Analysis of differential expression and identification of common DEGs

**Figure 1:**
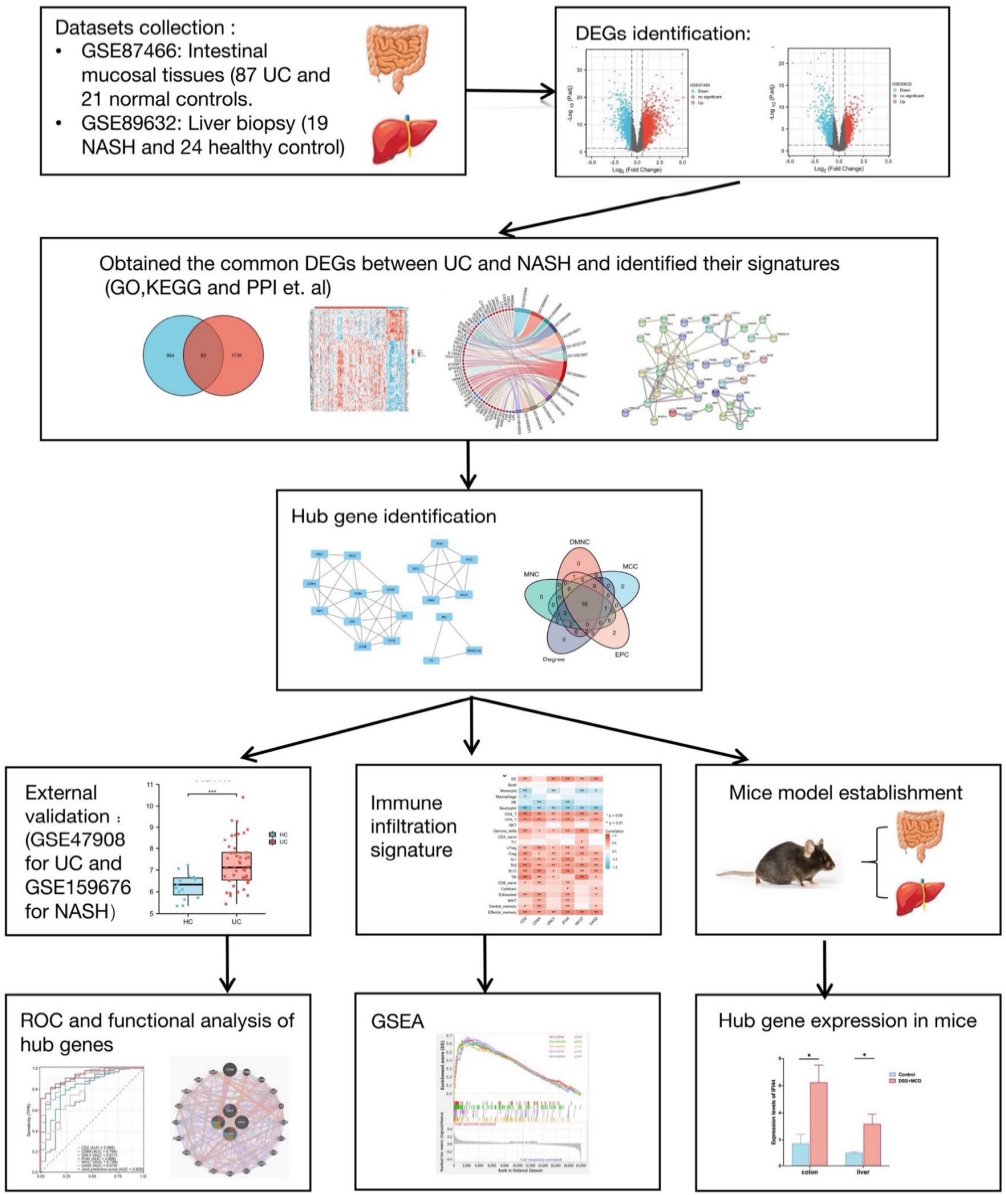
Schematic illustration of the overall workflow of this study.

**Figure 2:**
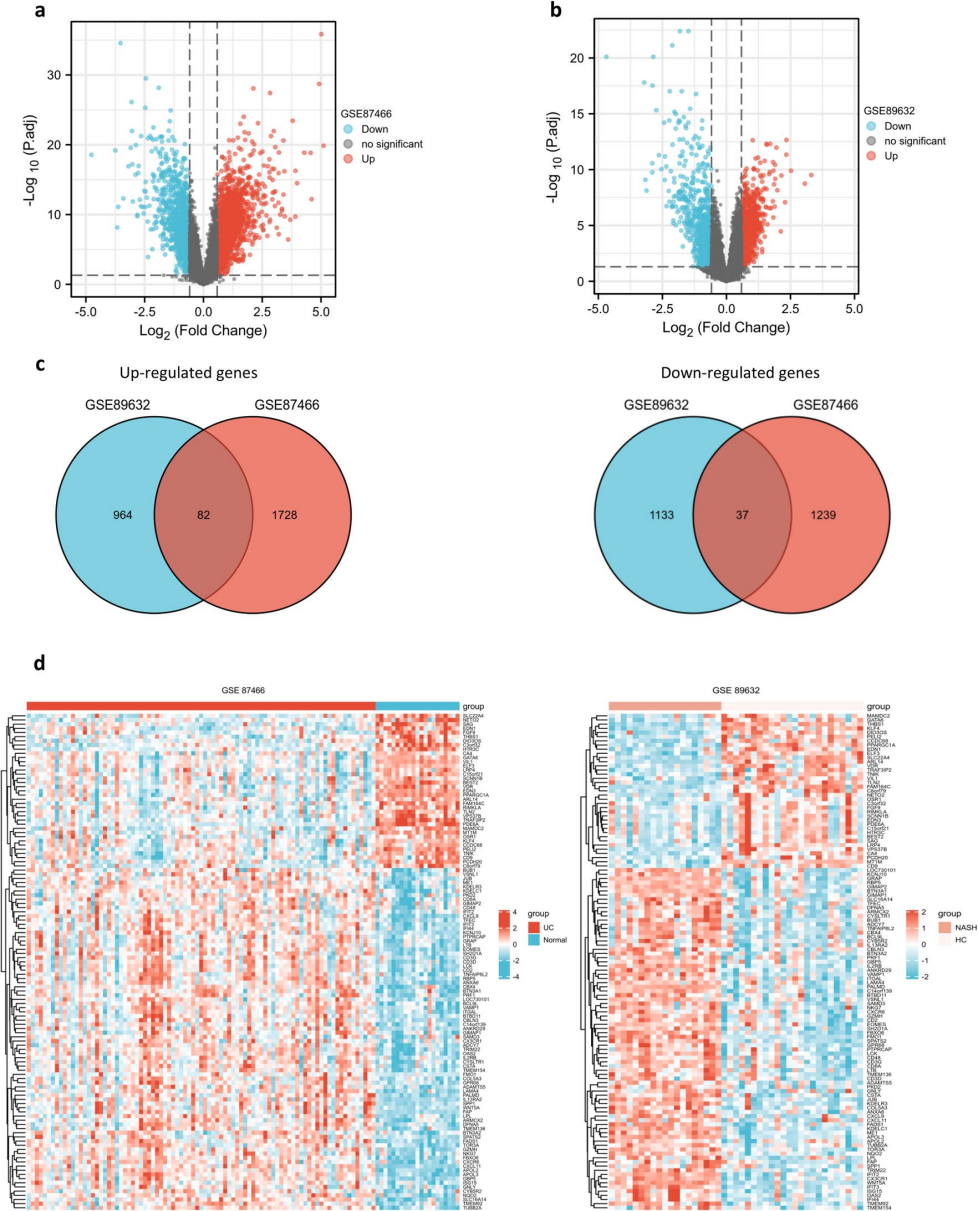
DEGs identification: (**a**) the volcano map of GSE87466 revealing DEGs between UC and Normal group. (**b**) The volcano map of GSE89632 revealing DEGs between NASH and HC group. (**c**) The same expression trends DEGs of the two databases. (**d**) The heat map of the same expression trends DEGs.

The overview of the study design is presented in the flow chart (Fig. 1). Standardized processing and DEG analysis were performed on the selected datasets. In GSE87466, 3086 DEGs were identified between UC patients and healthy controls, among which 1810 were upregulated and 1276 were downregulated (Fig. 2a). Meanwhile, 2216 DEGs were identified from NASH patients compared with the control group in dataset GSE89632, among which 1046 genes were upregulated and 1170 were downregulated (Fig. 2b). A total of 119 DEGs with the same expression trends were found in NASH and UC patients, of which 82 were upregulated and 37 were downregulated (Fig. 2c). Moreover, the heatmap shows the expression of common DEGs with the same trend in each sample (Fig. 2d).

### Functional annotation and pathway enrichment analysis

**Figure 3:**
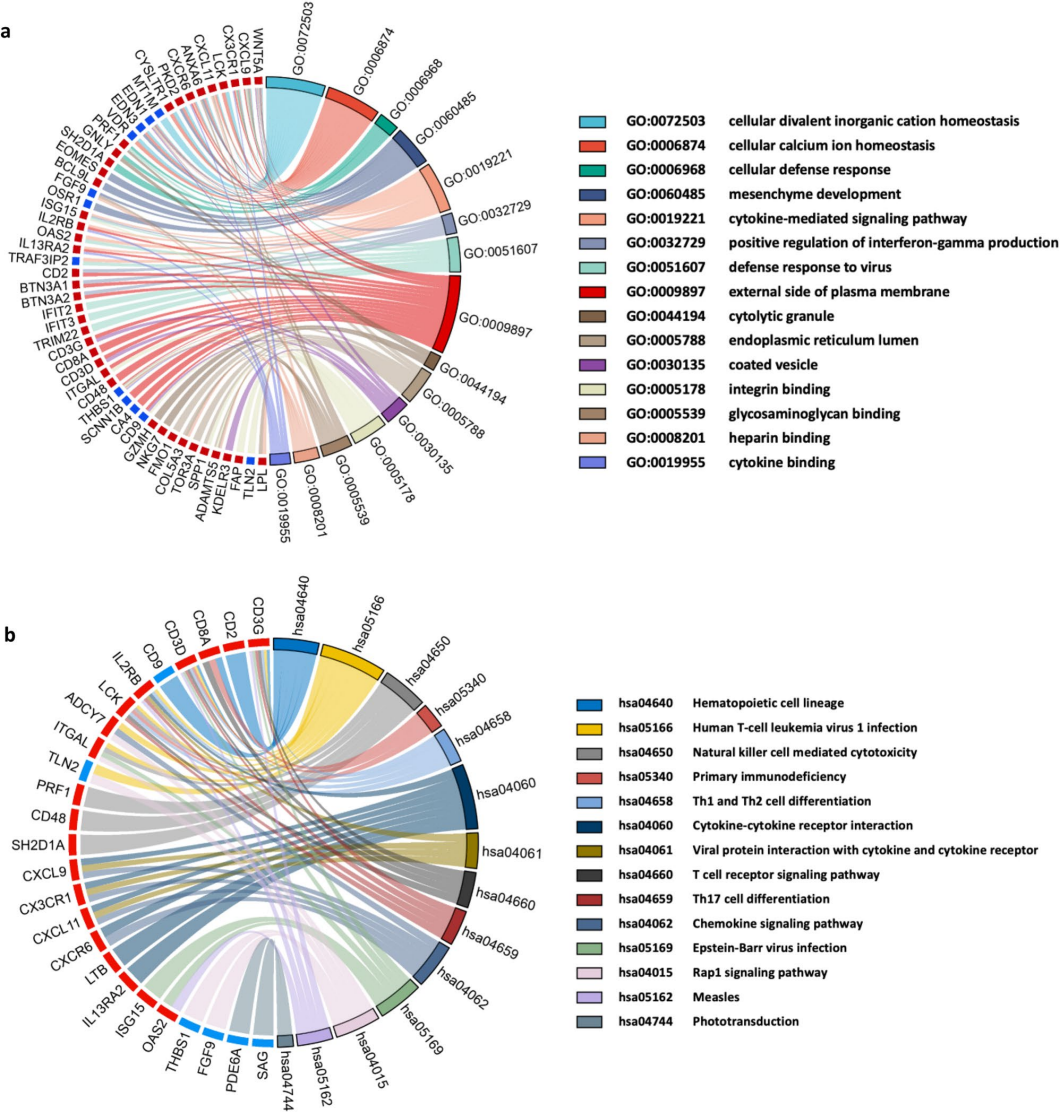
Functional annotation and pathway enrichment analysis: (**a**) the enrichment analysis results of GO on the same expression trends DEGs. (**b**) KEGG pathway analysis of the same expression trends DEGs.

We applied GO biological process enrichment and KEGG signaling pathway analyses to explore the common regulatory mechanism between UC and NASH. GO enrichment analysis showed that DEGs were mainly associated with cellular homeostasis, the cellular defense response, interferon-gamma secretion and the defense response to virus in terms of biological processes. In the cellular component category, genes were mainly enriched in the external side of the plasma membrane, endoplasmic reticulum lumen, cytolytic granules and coated vesicles. For molecular function, common genes were mainly enriched in integrin binding, heparin binding, cytokine binding and so on (Fig. 3a). Meanwhile, KEGG enrichment analysis results showed that DEGs were mainly focused on virus infection and immune response-related signaling pathways (Fig. 3b). Therefore, our enrichment results of gene function and pathway annotation showed that the common DEGs of UC and NASH are mainly involved in viral infection, inflammation and the immune response.

### PPI network construction and hub gene identification

**Figure 4:**
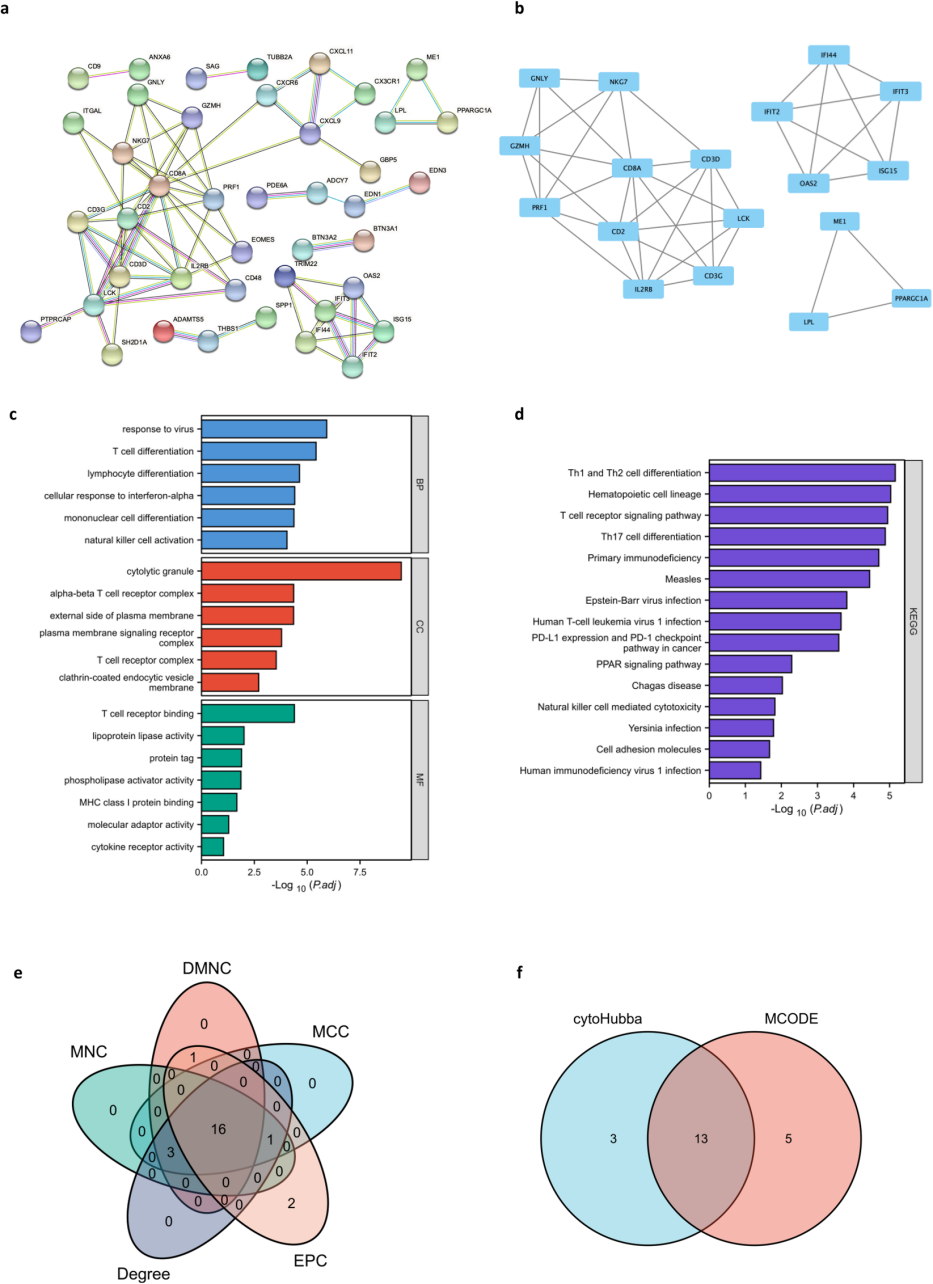
PPI network construction and hub gene identification: (**a**) PPI network mapped by STRING database. (**b**) Interconnected clusters of significant genes revealed by MCODE plugin. (**c**) GO analysis of the key nodes selected by MCODE. (**d**) KEGG pathway analysis of the key nodes selected by MCODE. (**e**) Overlapped the top 20 genes from 5 algorithms of cytoHubba. (**f**) Venn diagram of the key nodes selected by MCODE and intersection genes selected by cytoHubba.

To explore protein‒protein interactions and determine hub genes in these two diseases, we mapped the PPI network through the STRING database. As shown in Fig. 4a, we obtained a total of 42 nodes and 72 edges with PPI enrichment p values < 1.0e−16 when the interaction score was set as high confidence (> 0.7). Then, highly interconnected clusters were revealed by the Cytoscape plugin MCODE. According to the conditions set in the methods, three clusters were selected: Cluster 1 contained ten nodes and 29 edges with a score of 6.44; Cluster 2 contained five nodes and ten edges, with a score of 5; and Cluster 3 contained three nodes and three edges with a score of 3 (Fig. 4b). The nodes selected by MCODE were also examined by GO and KEGG enrichment analysis, which showed that they are also focused on virus infection and the inflammatory response, especially in T-cell-related pathways (Fig. 4c, d). Subsequently, we utilized cytoHubba, a plugin to define network topology, to identify the co-occurrence of important members in UC and NASH. The top 20 key target genes were ranked by the MCC, MNC, DMNC, EPC and Degree methods (Fig. 4e, Supplementary Table 1). The intersection of these top 20 genes from five different algorithms and from MCODE revealed 13 candidate hub genes through the intersection of the Venn diagram: CD8A, CD2, IL2RB, LCK, CD3D, CD3G, PRF1, IFIT3, OAS2, IFI44, GZMH, NKG7 and GNLY (Fig. 4f, Supplementary Table 2).

### External validation of the hub genes in independent datasets

**Figure 5:**
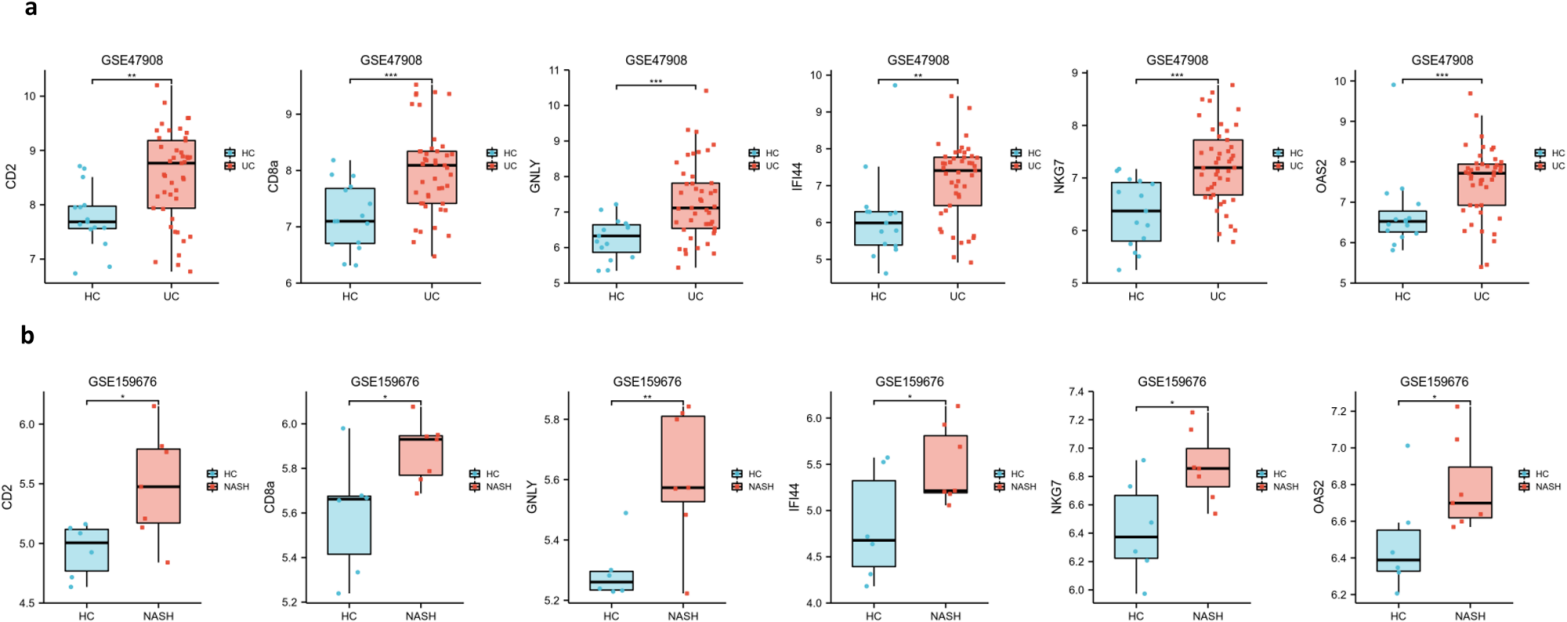
Validation of the hub genes: (**a**) the expression level of hub genes in GSE47908. (**b**) The expression level of hub genes in GSE159676.

**Figure 6:**
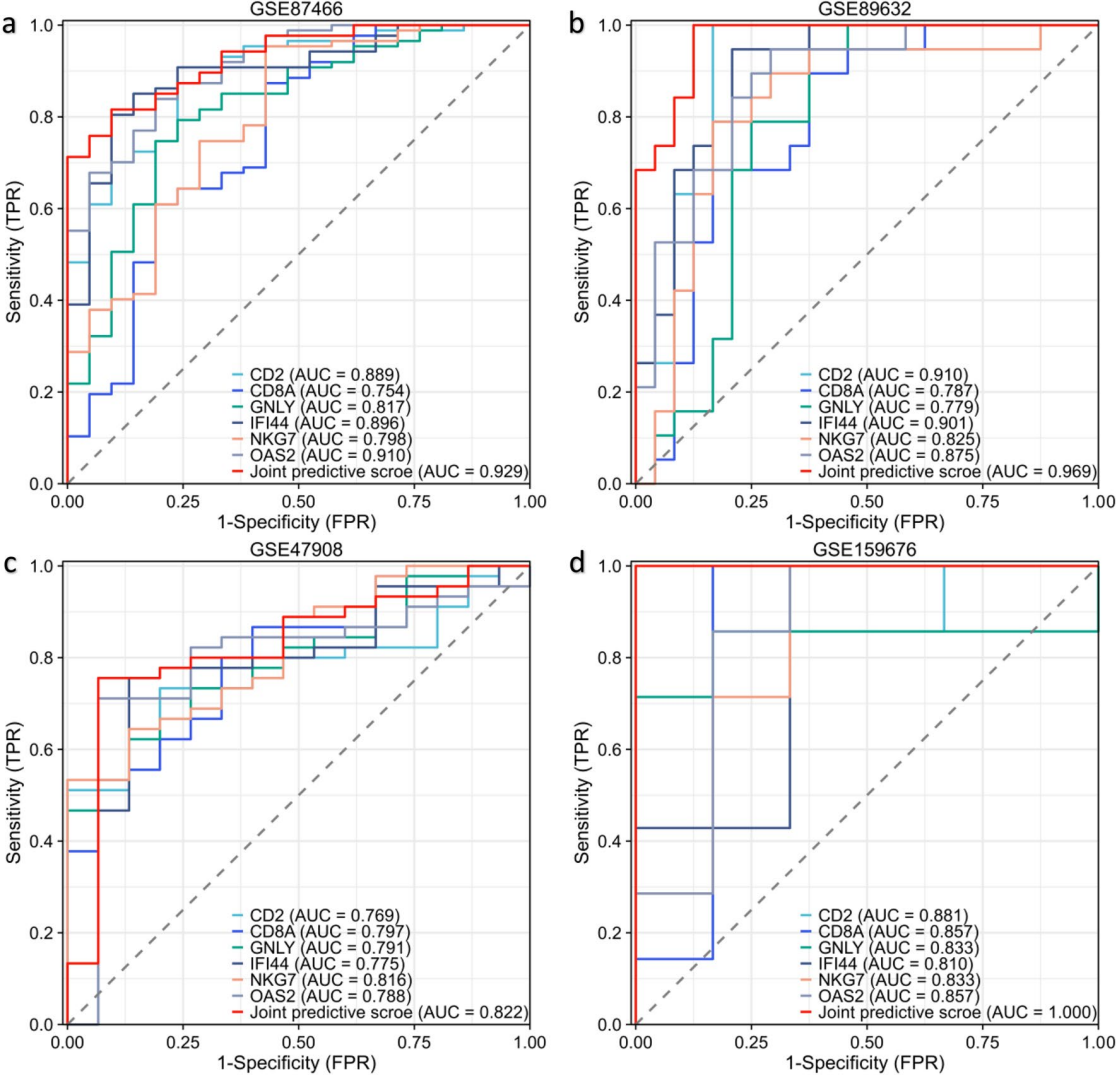
ROC curves of the hub genes: ROC curves were drawn to evaluate the accuracy of the hub genes and their joint predictive score of UC patients (**a,c**) as well as NASH (**b,d**).

To verify the efficacy of the core hub genes, we validated 13 candidate hub genes in two additional external datasets (GSE47908 for UC and GSE159676 for NASH). The results showed that compared with the control group, there were only six genes (CD2, CD8A, GNLY, IFI44, NKG7, and OAS2) with statistically significant differences in expression in the UC and NASH validated datasets (Fig. 5a, b). Then, we applied logistic regression analyses to estimate the joint predictive score of the six hub genes and constructed ROC curves to calculate the area under the ROC curve (AUC) to evaluate their performance. The results showed that the AUCs of CD2, CD8A, GNLY, IFI44, NKG7 and OAS2 in the four datasets were all greater than 0.7, and the joint predictive score had a better AUC after logistics analysis, which indicates that these six hub genes have good diagnostic efficacy for UC and NASH (Fig. 6).

### Functional analysis of hub genes and their interaction network

**Figure 7:**
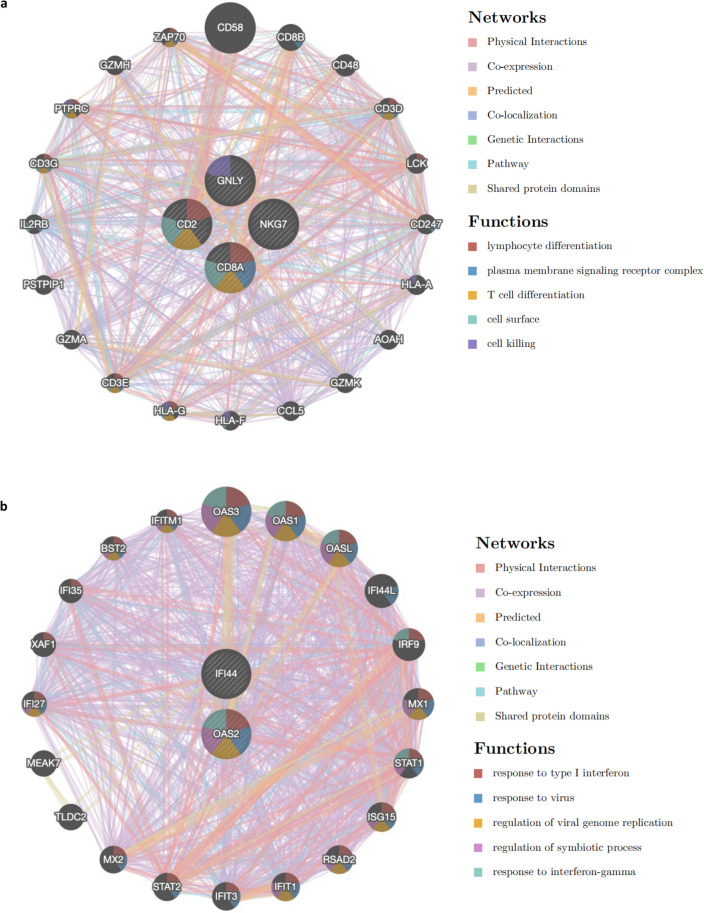
Functional analysis of hub genes and their interaction network: (**a**) the functions analysis of CD2, CD8A, GNLY, NKG7 and their interacting 20 genes. (**b**) The functions analysis of IFI44, OAS2 genes and its interacting genes.

Next, we performed functional analysis on hub genes and their interacting genes through GeneMANIA. The results demonstrated that the functions of the CD2, CD8A, GNLY and NKG7 genes and their interacting genes were mainly focused on lymphocyte differentiation, the plasma membrane signaling receptor complex, T-cell differentiation, the cell surface and cell killing (Fig. 7a). The functions of IFI44 and OAS2 genes and their interacting genes were focused on the response to type I interferon, response to virus, regulation of viral genome replication, regulation of symbiotic process and response to interferon-gamma (Fig. 7b). These results suggested that these 6 hub genes are closely related to inflammation and antiviral immunity.

### Immune infiltration signature identification

**Figure 8:**
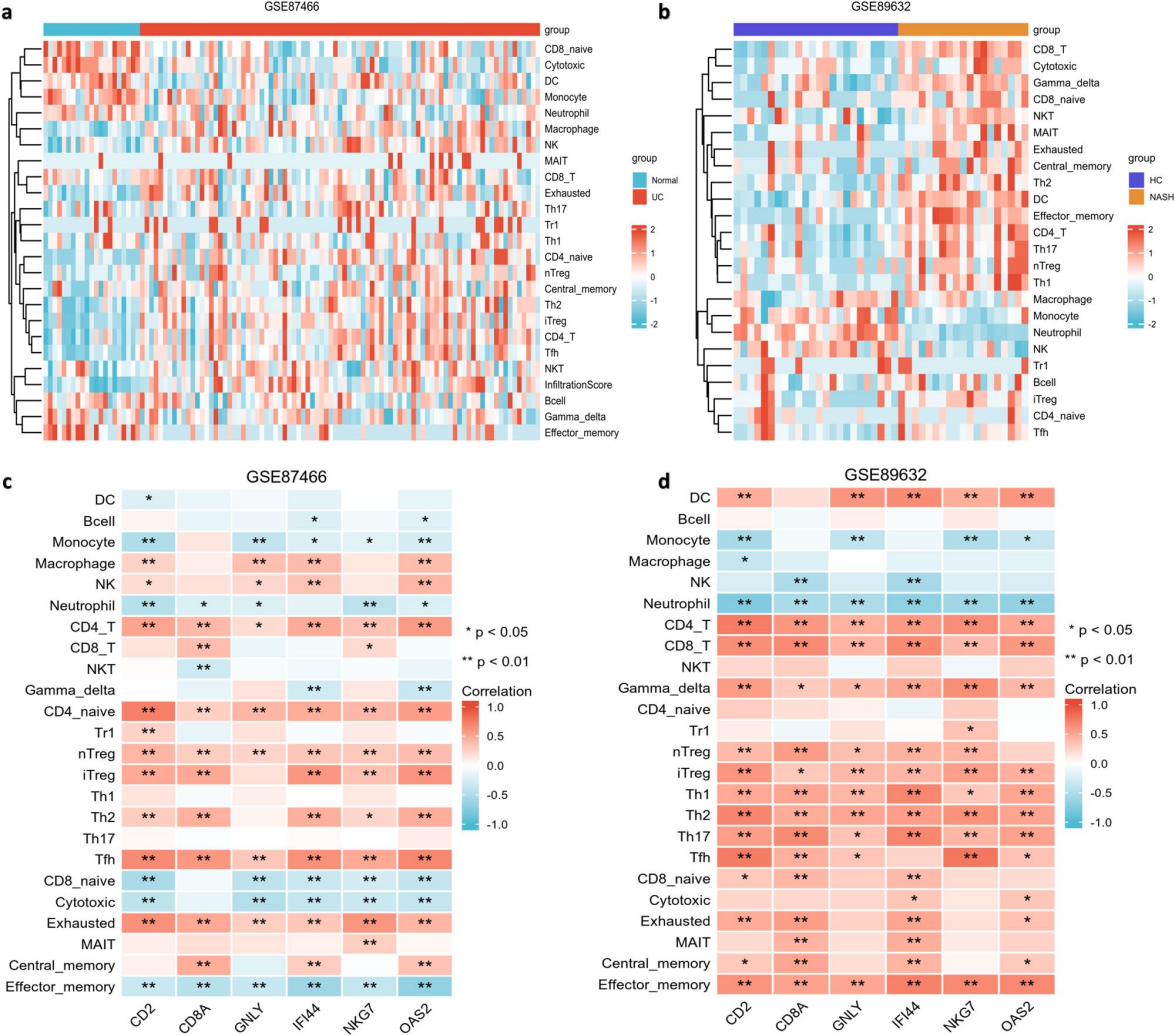
Immune infiltration signatures: (**a**) the heatmap of the immune cell infiltration between the normal group and UC group. (**b**) The heatmap of the immune cell infiltration between the healthy control and NASH group. (**c**) The correlation between the hub gene and the immune cell of GSE87466. (**d**) The correlation between the hub gene and the immune cell of GSE89632.

The ssGSEA algorithm in ImmuCellAI was used to determine the infiltration of immune cells in UC and NASH. The differences in immune cell abundance between the UC and normal groups (Fig. 8a) and between the NASH and healthy control groups (Fig. 8b) were evaluated. The abundances of CD4^+^T cells, CD8^+^T cells, T helper cells and regulatory T cells were extremely higher in both the UC group and NASH group. Then, we investigated the relationship between the six hub genes and 24 immune cell populations to determine whether the expression of these genes in UC and NASH was associated with a specific immune phenotype. As shown in Fig. 8c, d, hub genes were most correlated with CD4^+^T cells, CD4^+^naïve T cells, Treg cells and Th2 cells. CD8A and NKG7 also showed a positive correlation with CD8^+^T cells in the colon, and all six hub genes were significantly correlated with CD8^+^T cells in the liver. These results indicated that the six hub genes had notable relationships with T cells, natural killer cells and interferon type I, which suggests that severe immune imbalance occurs in UC and NASH.

### Hub gene examination by GSEA

**Figure 9:**
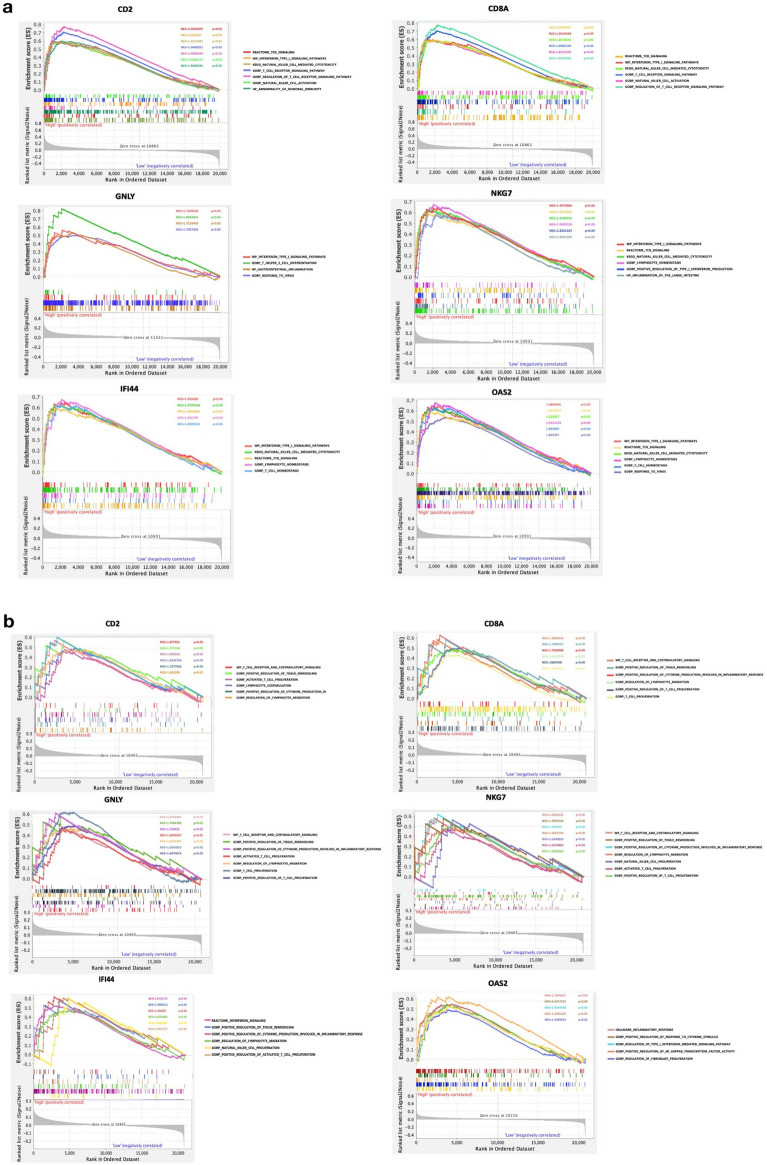
Gene set enrichment analysis: (**a**) merged enrichment of hub genes from GSE87466 of UC patients. (**b**) Merged enrichment of hub genes from GSE89632 of NASH patient.

GSEA was performed for the hub genes to elucidate the key regulated pathways between the high and low expression groups of hub genes involved in GSE87466 and GSE89632. The results showed that activation of pathways including T cell, natural killer cell and interferon type I signaling pathways was closely correlated with higher expression of hub genes. The inflammatory response and lymphocyte homeostasis are also remarkable (Fig. 9). These results indicate the potential functions and co-pathogenesis of the hub genes in UC and NASH.

### Mice model establishment and hub gene expression

**Figure 10:**
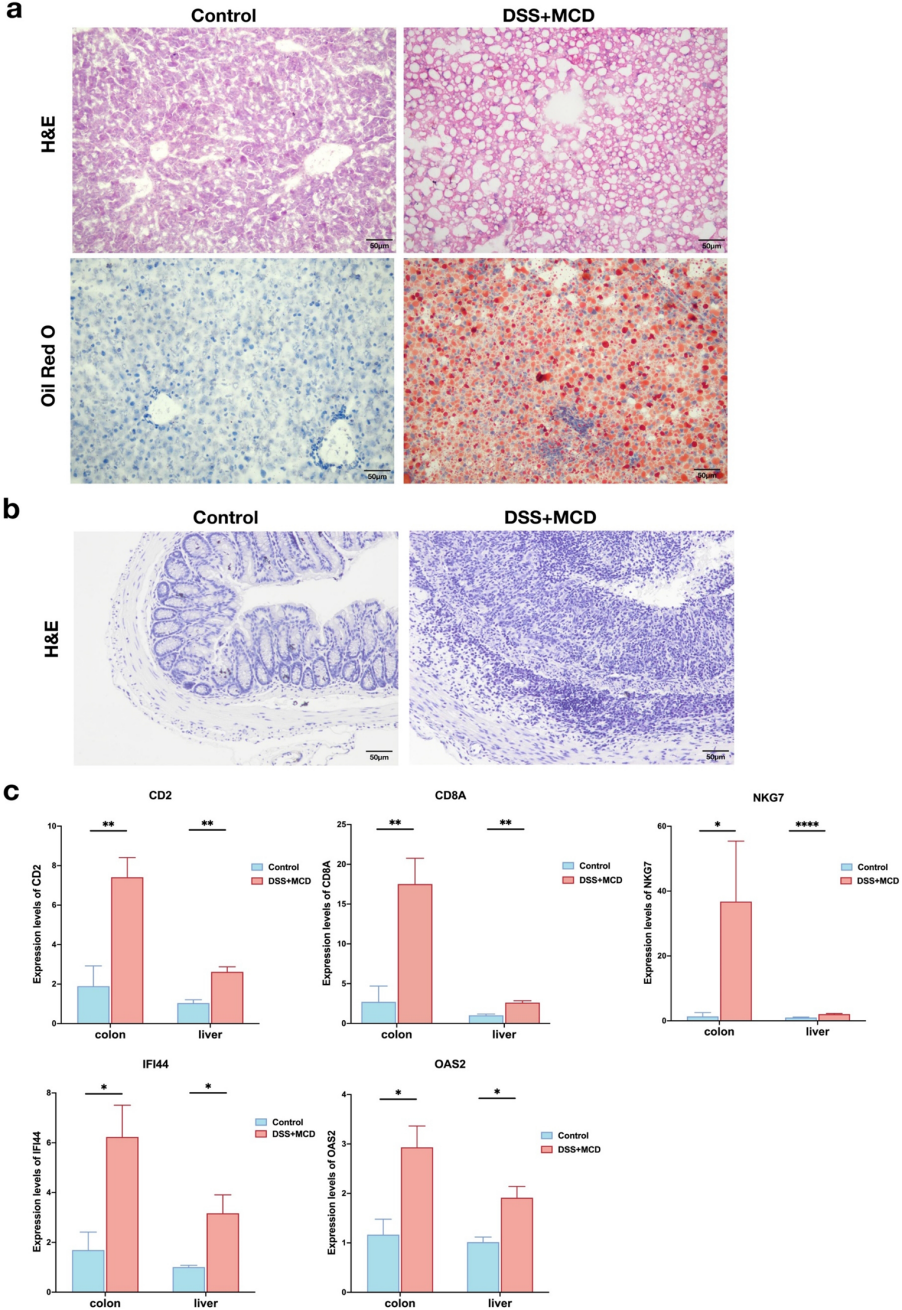
Histopathological staining and hub genes expression in mice models: (**a**) representative images of H&E and Oil-red O staining on liver sections (magnifications ×200); (**b**) representative images of H&E staining on colon sections (magnifications ×200); (**c**) relative expression of hub genes in colon and liver tissues.

We used DSS together with MCD to induce a mice model of UC combined with NASH. The results showed that the liver tissues of mice in the DSS + MCD group developed obvious hepatic steatosis, hepatocyte swelling and inflammatory cell infiltration compared with the control group. H&E staining of colonic tissues showed that the control group mice had intact colonic mucosa and clear glandular structures, while the mice in the DSS + MCD group had absent intestinal mucosa, shallow ulcer formation, glandular destruction, reduced goblet cells, and a large number of inflammatory cells infiltrating into the mucosal and submucosal layers (Fig. 10a, b).

Subsequently, we investigated the expression of hub genes in the UC combined NASH model, and since GNLY genes were not present in mice, we only validated the expression of CD2, CD8A, NKG7, IFI44 and OAS2 mRNA in the colon and liver tissues of mice. The results showed that the expression of hub genes in colon and liver tissues of mice in the DSS + MCD group were significantly higher than that in the control group, confirming that hub genes do have significant expression changes in the colon and liver tissue in the UC combined with NASH model, which may be important pathogenic factors in UC combined with NASH (Fig. 10c).

## Discussion

The pathogenesis of UC and NASH is still not completely clear and may be caused by complex interactions between polygenic susceptibility and multiple environmental factors. Hepatobiliary diseases, as one of the most common UC parenteral manifestations, may have the same pathogenesis or a co-pathogenesis. Therefore, identifying the core factors that cause the two diseases is important for early diagnosis and intervention. The main purpose of our study was to identify the common characteristics and to reveal potential therapeutic targets for UC and NASH to help develop biologics. In our study, we screened the RNA sequencing data of UC and NASH through the GEO database and identified 119 overlapping DEGs of the two diseases (82 upregulated and 37 downregulated) from the sequencing data. GO and KEGG enrichment analyses of these overlapping DEGs found that these genes were significantly enriched in immune, inflammatory and viral defense pathways, which is consistent with the current understanding of the progression of the two diseases. Subsequently, through analysis and verification, we finally identified six hub genes with significant differences between the two diseases, including CD2, CD8A, GNLY, IFI44, NKG7 and OAS2. These hub genes were validated in liver and intestinal in mice model of UC combined with NASH (except for GNLY, which is not present in rodents and still needs further verification in the future). ROC analysis showed that the six hub genes had great prediction and determination of the two diseases, and after logistics analysis, the combined index had a better AUC than that for any of the six hub genes alone, which indicates that these six hub genes have good diagnostic efficacy for UC and NASH.

Some studies show an increased prevalence of NAFLD in patients with IBD compared to the general population. In these cross-sectional studies, the prevalence of NAFLD in IBD ranged from 6.2 to 40%. In the recently published systematic reviews and meta-analyses, which contained 19 qualified studies including 5620 IBD patients, the overall pooled prevalence of NAFLD was 27.5% (95% CI, 20.7–34.2%). The heterogeneity between morbidity largely depends on different diagnoses and definitions of NAFLD^5,11–13^. A growing body of research has focused on the pathophysiological interaction between these two diseases. Preclinical studies have found that colitis induced by DSS can aggravate liver injury, inflammation, and fibrosis and even accelerate tumorigenesis in a mouse model of NASH^14–16^. In our previous study, we also explored the effects of UC on liver injury through the gut-liver axis and found that DSS-induced experimental colitis could aggravate CCL4-induced liver injury, inflammation and fibrosis by activating hepatic stellate cells and TLR4 signals^7^. Combined with clinical observations and preclinical trials, a variety of explanations for the increase in NASH in patients with IBD have been proposed, including metabolic syndrome (METS), length of disease, intestinal flora imbalance, intestinal barrier dysfunction and the effects of medications used to treat IBD^11,17^. However, due to the complexity of NASH and UC, the detailed co-pathogenesis of these two diseases remains unclear, and there are no current guidelines for screening or assessing NASH in patients with IBD^18^. It is still of great significance to find the connection and common mechanism between them.

Proteins encoded by the CD2 and CD8A genes are common surface glycoproteins. CD2 plays an important role in T cell activation, T or NK mediated cytolysis, apoptosis in activated peripheral T cells, and cytokine production by T cells^19^. It has been confirmed that genetic variation in CD2 is closely related to some immune diseases, and CD2 related signaling pathways have also been confirmed to be associated with virus clearance and autoimmune response enhancement^20,21^. CD8A encodes the α chain of the CD8 protein dimer, which is mainly involved in cellular immune defense and T-cell development^22^. CD8A can be used as a biomarker for the diagnosis and prognosis of many diseases, including tumors and inflammatory diseases^23,24^. Recent studies have shown that CD8A^+^IFNg^+^cNK cells are highly expressed in patients with active UC^25^. In addition, in high-fat high-fructose diet (HFHFD) induced NASH model, CD8A antibodies were used to reduce cytotoxic T lymphocytes (CTLs) numbers in liver tissue and induce biochemical and histologic attenuation of HFHFD induced NASH^26^.

NKG7 and GNLY are phenotypic marker genes closely related to NK cells and CTLs. NKG7 was initially considered to be an intrinsic membrane protein associated with cytotoxic granules, which promote the cytotoxic activity of T and NK cells^27^. Recent studies have found that expression of NKG7 may enhance the efficiency of CD8^+^T cells to form immune synapses with tumor targets and trigger cell death^28,29^. GNLY is a cytolytic and proinflammatory molecule that is secreted together with granzyme and perforin from the granules of human CTLs and NK cells and has extensive cytolytic effects on tumors and microorganisms. It also acts as a chemical attractor for T lymphocytes, monocytes and other inflammatory cells and activates the expression of many cytokines^30^. As GNLY is not present in rodents, it is unfortunate that we did not further validate its expression in our mice model. But GNLY has been found to play an important role in infection and immune-related diseases, such as osteoarthritis, psoriasis and chronic viral hepatitis^31–33^. Currently, human GNLY gene has been constructed into transgenic mice models and effectively used in experimental investigations. The application of h-GNLY transgenic mice will play an important role in the future exploration of GNLY related function as well as in NASH combined with UC. Therefore, NKG7 and GNLY are gradually being regarded as targets for the treatment of inflammation or tumors through inhibition or stimulation of their expression, and their synthetic forms are being developed as novel drugs for new diagnostics and treatments for a variety of diseases^34^.

The causal links between viral infection and autoimmune diseases have been studied, although they are still elusive. Infectious factors may induce and promote the progression of immune diseases, among which interferon (IFN)-related immune responses are particularly important, which may represent the molecular link between environmental triggers and critical immune molecules^35^. OAS2 and IFI44 are typical interferon-stimulated genes (ISGs). IFI44 is a type I IFN induction protein that has been shown to be upregulated in different viral infections and immune-related diseases. However, some studies reached the opposite conclusion, suggesting that IFI44 may be a negative feedback regulator of the virus-induced IFN response^36,37^. OAS2 is an essential protein in the innate immune response against viral infection regulated by IFN. It has been confirmed that the expression of OAS2 is increased in the colonic mucosa of patients with active UC and plays an important role in hepatic steatosis and HCV infection^38,39^. The expression and function of IFI44 and OAS2 still need further exploration and experimental verification, especially in chronic inflammatory and immune diseases.

In our study, CD2, CD8A, GNLY and NKG7, as a cluster of core functional genes, were subjected to functional analysis with their interacting genes combined with the results of Cytoscape and GeneMANIA, and we found that they were mainly focused on lymphocyte differentiation, the plasma membrane signaling receptor complex, T-cell differentiation, the cell surface and cell killing. The functions of IFI44 and OAS2 genes and their interacting genes were focused on the response to type I interferon, the response to virus, regulation of viral genome replication, regulation of symbiotic process and the response to IFN-γ. This is also consistent with our knowledge of these genes individually and the development of these two diseases, that is, infection, inflammation and immunity, which play important roles in the pathogenesis of NASH and UC.

Immunity and inflammation are not only important defense mechanisms against infection and injury but also important aspects of tissue repair^40^. However, persistent inflammation can damage tissue and ultimately be harmful to the host. With the progress of the investigation of these two diseases, the gut-liver axis has received increasing attention. The concept of the gut-liver axis was first proposed by Marshall in 1998. The gut and liver share common embryological origins, and they are intrinsically linked in anatomical and biological functions. In the healthy state, homeostasis of the immune system is a fine balance between the gut and liver, and links between responses in the gut and the liver are through the biliary tract, portal vein and circulatory system in the gut-liver axis^6,41^. UC is usually accompanied by intestinal flora disorder and intestinal barrier function damage, and bacteria and various flora metabolites enter the host circulation and liver through the enterohepatic axis. A number of immune cells in the liver resist proinflammatory factors from the intestinal tract, which may lead to excessive activation of immune cells and further promote liver injury, inflammatory reactions and fibrosis, leading to the formation and development of NASH. In our study, six hub genes involved in NASH and UC were screened, which mainly played an important role in infection, inflammation and immunity. In addition, the six hub genes were examined for associations with the levels of 24 kinds of immune cells in NASH and UC. The results showed that all six hub genes were positively correlated with effector T cells, T helper cells and T follicular helper cells, which further confirmed the important influence of the six hub genes on the development and differentiation of T cells and the antiviral and immune effects in the two diseases. Further verification by GSEA also confirmed that the expression of the six hub genes plays a role in inflammatory immunity and viral infection, especially in regards to T-cell function, in both diseases.

Current studies mainly evaluate and explore the interaction between UC and NASH and possible mechanisms by clinical observation, endoscopy and imaging examinations. Our study explored these two diseases at the gene level and found potential biochemical markers and related functions that may affect the clinical outcome of UC coexistence with NASH. However, some limitations of this study need to be noted. First, the sequencing data of UC and NASH were derived, and the sample was limited and sequenced from different platforms. If liver and intestinal mucosa samples of UC patients coexistence with NASH can be obtained in the future, the results will be more accurate. Second, we have constructed a mice model of UC combined with NASH and performed preliminary assays of the selected hub genes in mice, but we have not explored the functions of these genes, and we will further investigate the functions of these hub genes and their encoded proteins in vivo and in vitro experiments.

## Conclusions

This study elucidates CD2, CD8A, GNLY, IFI44, NKG7 and OAS2 as the core genes of UC and NASH. These genes may play important roles in disease pathogenesis by participating in immune, inflammatory and antiviral effects. Our study provides some potential biomarkers and targets, which provides new insights for further exploring the molecular mechanism and new therapeutic drugs for UC coexistence with NASH.

### Supplementary Information

Supplementary Table 1.

Supplementary Table 2.

## Data Availability

The results we analyzed in our paper were based data from GEO public database. The data produced by mice model and materials are available from the corresponding author upon reasonable request.

## Abbreviations

AUC: Area under the ROC curve
BP: Biological process
CC: Cellular component
DEGs: Differentially expressed genes
DMNC: Density of maximum neighborhood component
DSS: Dextran sulfate sodium
EPC: Edge percolated component
FCs: Fold changes
GEO: Gene Expression Omnibus
GO: Gene Ontology
GSEA: Gene set enrichment analysis
IBD: Inflammatory bowel disease
KEGG: Kyoto Encyclopedia of Genes and Genomes
MCC: Maximal clique centrality
MCD: Methionine choline deficiency die
MCODE: Molecular complex detection
MCS: Methionine and choline sufficient diet
MF: Molecular function
MNC: Maximum neighborhood component
NAFLD: Nonalcoholic fatty liver disease
NASH: Nonalcoholic steatohepatitis
PPI: Protein–protein interaction
ssGSEA: Single sample gene set enrichment analysis
STRING: Search Tool for the Retrieval of Interacting Genes Database
UC: Ulcerative colitis
