# Supplementary material for: Shared biomarkers and immune cell infiltration signatures in ulcerative colitis and nonalcoholic steatohepatitis
Source: Sci Rep. 2023 Oct 28;13:18497. doi: 10.1038/s41598-023-44853-6 (PMC10613305; doi:10.1038/s41598-023-44853-6)
Supplement: Supplementary file 1 — Supplementary Table 1. [file 41598_2023_44853_MOESM1_ESM.docx]

**Supplementary Table 1**

**The top 20 hub genes rank in cytoHubba.**

| MCC | DMNC | MNC | Degree | EPC |
| --- | --- | --- | --- | --- |
| CD8A | CD3G | CD8A | CD8A | CD8A |
| CD2 | ISG15 | CD2 | CD2 | CD2 |
| IL2RB | IFIT2 | IL2RB | LCK | LCK |
| LCK | GNLY | LCK | IL2RB | IL2RB |
| CD3D | IFIT3 | CD3D | CD3D | PRF1 |
| CD3G | OAS2 | PRF1 | PRF1 | CD3D |
| PRF1 | IFI44 | CD3G | CD3G | CD3G |
| IFIT3 | GZMH | IFIT3 | IFIT3 | GZMH |
| OAS2 | IL2RB | OAS2 | OAS2 | NKG7 |
| IFI44 | LCK | IFI44 | IFI44 | GNLY |
| GZMH | TRIM22 | GZMH | GZMH | EOMES |
| NKG7 | EOMES | NKG7 | NKG7 | CD48 |
| ISG15 | CD48 | ISG15 | CXCL9 | CXCL9 |
| IFIT2 | NKG7 | IFIT2 | ISG15 | ITGAL |
| GNLY | CD3D | GNLY | IFIT2 | SH2D1A |
| CXCL9 | PRF1 | CXCL9 | GNLY | CXCR6 |
| TRIM22 | CD2 | TRIM22 | TRIM22 | CXCL11 |
| EOMES | CD8A | EOMES | EOMES | IFI44 |
| CD48 | CXCL11 | CD48 | CD48 | IFIT3 |
| CXCL11 | CXCR6 | CXCL11 | CXCL11 | OAS2 |
