# Supplementary material for: Shared biomarkers and immune cell infiltration signatures in ulcerative colitis and nonalcoholic steatohepatitis
Source: Sci Rep. 2023 Oct 28;13:18497. doi: 10.1038/s41598-023-44853-6 (PMC10613305; doi:10.1038/s41598-023-44853-6)
Supplement: Supplementary file 2 — Supplementary Table 2. [file 41598_2023_44853_MOESM2_ESM.docx]

**Supplementary Table 2**

**The details of candidate hub genes**

| **Gene** | **Full name** | **Function** |
| --- | --- | --- |
| CD2 | CD2 molecule | The protein encoded by this gene is a surface antigen found on all peripheral blood T-cells and interacts with LFA3 (CD58) on antigen presenting cells to optimize immune recognition. |
| CD8A | CD8 subunit alpha | The CD8 antigen is a cell surface glycoprotein found on most cytotoxic T lymphocytes that mediates efficient cell-cell interactions within the immune system. The CD8 antigen acts as a coreceptor with the T-cell receptor on the T lymphocyte to recognize antigens displayed by an antigen presenting cell in the context of class I MHC molecules. This gene encodes the CD8 alpha chain. |
| IL2RB | interleukin 2 receptor subunit beta | The interleukin 2 receptor, which is involved in T cell-mediated immune responses. The protein encoded by this gene represents the beta subunit and is a type I membrane protein. |
| LCK | LCK proto-oncogene, Src family tyrosine kinase | This gene is a member of the Src family of protein tyrosine kinases (PTKs). The encoded protein is a key signaling molecule in the selection and maturation of developing T-cells. The protein localizes to the plasma membrane and pericentrosomal vesicles, and binds to cell surface receptors, including CD4 and CD8, and other signaling molecules. |
| CD3D | CD3 delta subunit of T-cell receptor complex | The protein encoded by this gene is part of the T-cell receptor/CD3 complex (TCR/CD3 complex) and is involved in T-cell development and signal transduction. The encoded membrane protein represents the delta subunit of the CD3 complex. Defects in this gene are a cause of severe combined immunodeficiency autosomal recessive T-cell-negative/B-cell-positive/NK-cell-positive (SCIDBNK). |
| CD3G | CD3 gamma subunit of T-cell receptor complex | The protein encoded by this gene is the CD3-gamma polypeptide, which together with CD3-epsilon, -delta and -zeta, and the T-cell receptor alpha/beta and gamma/delta heterodimers, forms the T-cell receptor-CD3 complex. This complex plays an important role in coupling antigen recognition to several intracellular signal-transduction pathways. Defects in this gene are associated with T cell immunodeficiency. |
| PRF1 | perforin 1 | This gene encodes a protein with structural similarities to complement component C9 that is important in immunity. This protein forms membrane pores that allow the release of granzymes and subsequent cytolysis of target cells. |
| GNLY | granulysin | The product of this gene is a member of the saposin-like protein (SAPLIP) family and is located in the cytotoxic granules of T cells, which are released upon antigen stimulation. This protein is present in cytotoxic granules of cytotoxic T lymphocytes and natural killer cells. |
| NKG7 | natural killer cell granule protein 7 | Predicted to be integral component of plasma membrane and to be active in plasma membrane. |
| IFI44 | interferon induced protein 44 | Predicted to be involved in immune response and to act upstream of or within response to bacterium. |
| OAS2 | 2'-5'-oligoadenylate synthetase 2 | This gene encodes a member of the 2-5A synthetase family, essential proteins involved in the innate immune response to viral infection. The encoded protein is induced by interferons and uses adenosine triphosphate in 2'-specific nucleotidyl transfer reactions to synthesize 2',5'-oligoadenylates (2-5As). These molecules activate latent RNase L, which results in viral RNA degradation and the inhibition of viral replication. |
| IFIT3 | interferon induced protein with tetratricopeptide repeats 3 | Enables identical protein binding activity. Involved in negative regulation of apoptotic process; negative regulation of cell population proliferation; and response to virus. |
| GZMH | granzyme H | This gene encodes a member of the peptidase S1 family of serine proteases. This protein is reported to be constitutively expressed in the NK (natural killer) cells of the immune system and may play a role in the cytotoxic arm of the innate immune response by inducing target cell death and by directly cleaving substrates in pathogen-infected cells. |
